# Abdominal ultrasound-scanning versus non-contrast computed tomography as screening method for abdominal aortic aneurysm – a validation study from the randomized DANCAVAS study

**DOI:** 10.1186/s12880-017-0186-8

**Published:** 2017-02-14

**Authors:** Mads Liisberg, Axel C. Diederichsen, Jes S. Lindholt

**Affiliations:** 1 Department of Cardiothoracic and Vascular Surgery, Odense University Hospital, Cardiovascular Centre of Excellence (CAVAC), Sdr. Boulevard 29, Afd. T - Forskerreden, 5000 Odense C, Denmark; 2 Elitary Research Centre of Individualised Treatment of Arterial Diseases (CIMA), Odense University Hospital, Odense C, Denmark; 3 Department of Cardiology, Odense University Hospital, Odense C, Denmark; 4 OPEN, Odense Patient data Explorative Network, Odense University Hospital, Odense C, Denmark

## Abstract

**Background:**

Validating non-contrast-enhanced computed tomography (nCT) compared to ultrasound sonography (US) as screening method for abdominal aortic aneurysm (AAA) screening.

**Methods:**

Consecutively attending men (*n* = 566) from the pilot study of the randomized Danish CardioVascular Screening trial (DANCAVAS trial), underwent nCT and US examination. Diameters were measured in outer-to-outer fashion. Sensitivity and specificity were done testing each modality against each other as reference standard. Measurements were tested for correlation, variance in diameters, and mean differences were tested using paired *t*-test.

**Results:**

Due to logistics, 533 underwent *both* nCT and US. In four patients, aortae could not be visualized with US, and two of these had an AAA (>30 mm) as diagnosed by nCT. Using nCT 30 (5.7%, 95% CI: 4.2;7.5%) AAA were found. US failed to detect 9 of these, but diagnosed 3 other cases, resulting prevalence by US was 4.5% (95% CI: 3.0;6.6%). Additionally, 5 isolated iliac aneurysms (≥20 mm) (0.9%, 95% CI: 0.3;2.2%) were discovered by nCT.

US performed reasonably, with sensitivity ranging from 57.1–70.4%, specificity however, ranged higher 99.2–99.6%. Comparably nCT performed with sensitivity ranging from 82.6–88.9%, nCTs specificity however ranged from 97.7–98.4%. Analysis showed good correlations with no tendency to increasing variance with increasing diameter, and no significant differences between nCT and US with means varying slightly in both axis.

**Conclusions:**

nCT seems superior to US concerning sensitivity, and is able to detect aneurysmal lesions not detectable with US. Finally, the prevalence of AAA in Denmark seems to remain relatively high, in this small pilot study group.

## Background

Screening for abdominal aortic aneurysm (AAA) based upon abdominal aortic ultrasound sonography (US) has proven beneficial, cost-effective, which partly is the reason why US-based screening programs have been implemented in several countries [1–3]. However as reported by the MASS trial [3], AAA related deaths do occur years after screening programs finding normal aortas in the attenders. This might be prevented by rescreening, although intervals for rescreening in normal aortas have yet to be established. Following this, the reduced AAA specific mortality by screening is only about 50%, which contrasts with reported attendance rates close to 80% [4]. The specific causes are unknown - it could be, that those in high risk do not attend, or down to false negative findings, incidental development, a combination, or mistaken recorded cause of death.

Today two modalities are utilized to assess the infrarenal aortic diameter (IAD) to diagnose AAA, namely US and computed tomography (CT), each with their own benefits, and drawbacks.

As a screening modality for AAA, US has become accepted, because it is easy to operate, cheap and with an estimated sensitivity and specificity close to 100% [5]. This however, was based upon the size distribution in the population, and observed intervariation of US measurements. In reality, US has never been validated as a screening modality for AAA, it has only been validated when AAA was present, and even when present with significant interobserver variability [6–8]. Adding to this, some infrarenal aortas are difficult to visualize due to intestinal gas and/or adiposity [9].

Using non-contrast CT scanning as an alternative screening method for AAA might be more reliable, and offer other screening potentials as coronary calcifications, thoracic- and iliac lesions. Because CT scanners are becoming widely available and perform better with each iteration, while using less radiation due to modern iterative reconstruction algorithms, effectively enabling CT to be a valid screening modality.

Contrast enhanced CT-scans are known to be more precise, probably with 100% sensitivity and specificity, but have not been tested as a screening tool. Additionally, it would expose the examined individuals not only for radiation, but potential nephrotoxic contrast. Contrast enhanced CT-scans are not widely available, time consuming and thus expensive, making it a less rational screening modality.

Nearly half the population in the Western world dies due to cardiovascular diseases (CVD), mainly due to ischemic heart disease. Focusing on traditionally risk markers like hypertension, hypercholesterolemia and diabetes screening and intervention have been tested in randomized setups, and proven insufficient [10]. The question is whether detection of asymptomatic arterial lesions could lead to a better risk stratification and intervention. Low dose non-contrast-enhanced cardiac CT scan quantifying the degree of coronary arterial calcification, and has been proven to be one of the best predictors of future cardiac events [11, 12], and might be the tool for future screening and intervention. If such a scan is expanded to include the chest and abdomen, thoracic as well as abdominal aortic aneurysms would be exposed, but the question is whether infrarenal aorta will be sufficiently visualized. This question arises from the modern low dosage scans used in cardiac CT which might not visualize the infrarenal aorta sufficiently.

Consequently, in the pilot study of the randomized Danish CardioVascular Screening trial (DANCAVAS trial) men underwent screening for AAA by both US and non-contrast-enhanced CT scanning (nCT) [13, 14]. The aim of this study, is to validate nCT as a comparable modality to US in a AAA screening setting.

## Methods

### Design

Population based cross-sectional study within a population based multicenter randomized screening trial. All Danish citizens are given a unique civil registration number at birth, with which we are able to track all their interactions with the Danish health institutions (e.g. hospital admissions, drug prescriptions etc.). Through this registry 45.000 men will be randomly selected based on their age, and geographic location, to correspond to our screening sites. A third of the selected men will be invited to our cardiovascular screening program, whilst the remaining two thirds will be followed through the registries. There are no exclusion criteria for the participants in this study. This article will only be analyzing data from primary attenders the pilot study, consisting of 956 invitees, of which 566 attended primarily.

### Participants

The DANCAVAS trial is an ongoing multicenter trial with Danish screening sites in Odense, Svendborg, Vejle and Silkeborg. Ethical approval was obtained by the Southern Denmark Region Committee on Biomedical Research Ethics (S-20140028) and the Data Protection Agency, and registered in ISRCTN (DOI 10.1186/ISRCTN12157806) [13]. The study protocol was reviewed and approved by the institutional review board, all participants were given written and oral information about the study, and written consent was obtained from each participant.

The primary aim is to investigate whether combined advanced cardiovascular screening will prevent death and cardiovascular events, and whether the likely health benefits are cost effective.

One-third of 45.000 will be invited a screening examinations at one of the 4 locations. The screening will include: (1) nCT scan to detect coronary artery calcification above the corresponding age median, and aortic/iliac aneurysms, (2) Brachial and ankle blood pressure index to detect peripheral arterial disease and hypertension, (3) an assessment of the CT monitored heart rhythm to detected atrial fibrillation, and (4) a measurement of the cholesterol and plasma glucose levels. Up-to-date cardiovascular preventive treatment is recommended in case of positive finding. Positive AAA findings is defined as infra renal aortic diameter ≥30 mm, and iliac aneurysms are defined as ≥20 mm.

In Odense, men aged 65–74 were consecutively invited to participate in the DANCAVAS *pilot* screening program in the autumn 2014, with *no exclusion criteria*. In total, 956 were invited and 566 attended initially when this validation study took place.

### Imaging

Medical students, received training by an experienced vascular surgeon, before being allowed to evaluate participants used a GE Logiq E9 with a C1-5-D or C1-6-D transducer to perform all ultrasound abdominal aortic measurements. Using the cinematic function, the maximal systolic outer-to-outer diameter was measured in the anterior-posterior (AP) and transverse plane [15, 16]. The US examinations were blinded to the results from the nCT examinations carried out consecutively, and vice versa.

Low dose nCT were performed with a Siemens Somatom Definition Flash: spiral scan with a pitch of 3.2 (Flash), 100 kV tube voltage, 90 mAs, collimation of 128 x 0.6 mm, Safire 3 and slice thickness 5 mm from the thoracic aorta, to the common femoral arteries. Trained radiographers, using Siemens Syngo.via, evaluated the resulting CT-images. In case of an obvious aneurysm, the diameters were measured outer to outer, measurements were in the axis of the aorta for both AP/transverse planes. In case of no aneurysms the outer to outer dimensions of the abdominal aorta was measured in a transversal and an anterior-posterior plane just above the bifurcation of the aorta. Diameters of the iliac arteries were noted in case of aneurysm.

#### Statistical analysis

Data was initially merged in a 2x2 table (Tables 1A-C) and sensitivity and specificity was calculated, using each method as reference standard for the other. Sensitivity and specificity as well as predictive values are presented in percentages for ease of interpretation, their confidence intervals are ‘exact’ Clopper-Pearson confidence intervals.

**Table 1:**
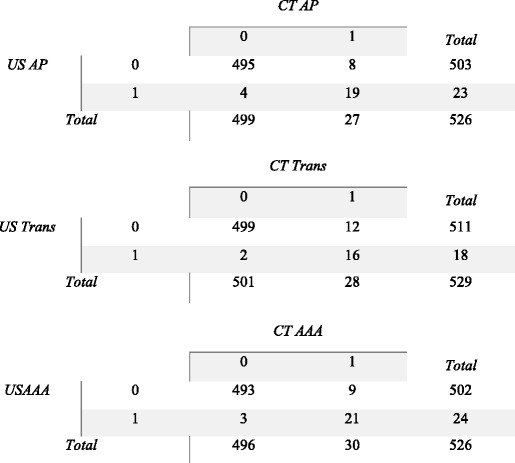
A-C, Title: Cross tabulation of results used for sensitivity calculations

Legend: US/CT AP – 0 denotes an AP diameter of <30 mm; 1 denotes an AP diameter of >30 mm

US/CT Trans - 0 denotes a Transverse diameter of <30 mm; 1 denotes a Transverse diameter of >30 mm

US/CT AAA – 0 denotes any US measurements <30 mm; 1 denotes any measured diameter >30 mm

The data was mainly analyzed as suggested by Bland and Altman [17]. First data was examined for normal-distribution, this was found to be true, although diameters slightly shifted to the left graphically. Secondly, data were examined by plotting the results from nCT against US. Systematic differences between the two methods were tested by paired *t*-test. Statistical analysis was carried out using SPSS 22 (IBM Corp. Released 2013. IBM SPSS Statistics for Windows, Version 22.0. Armonk, NY: IBM Corp.) and Stata 14 (StataCorp. 2015. Stata Statistical Software: Release 14. College Station, TX: StataCorp LP.).

## Results

### Visibility and prevalence

**Fig. 1:**
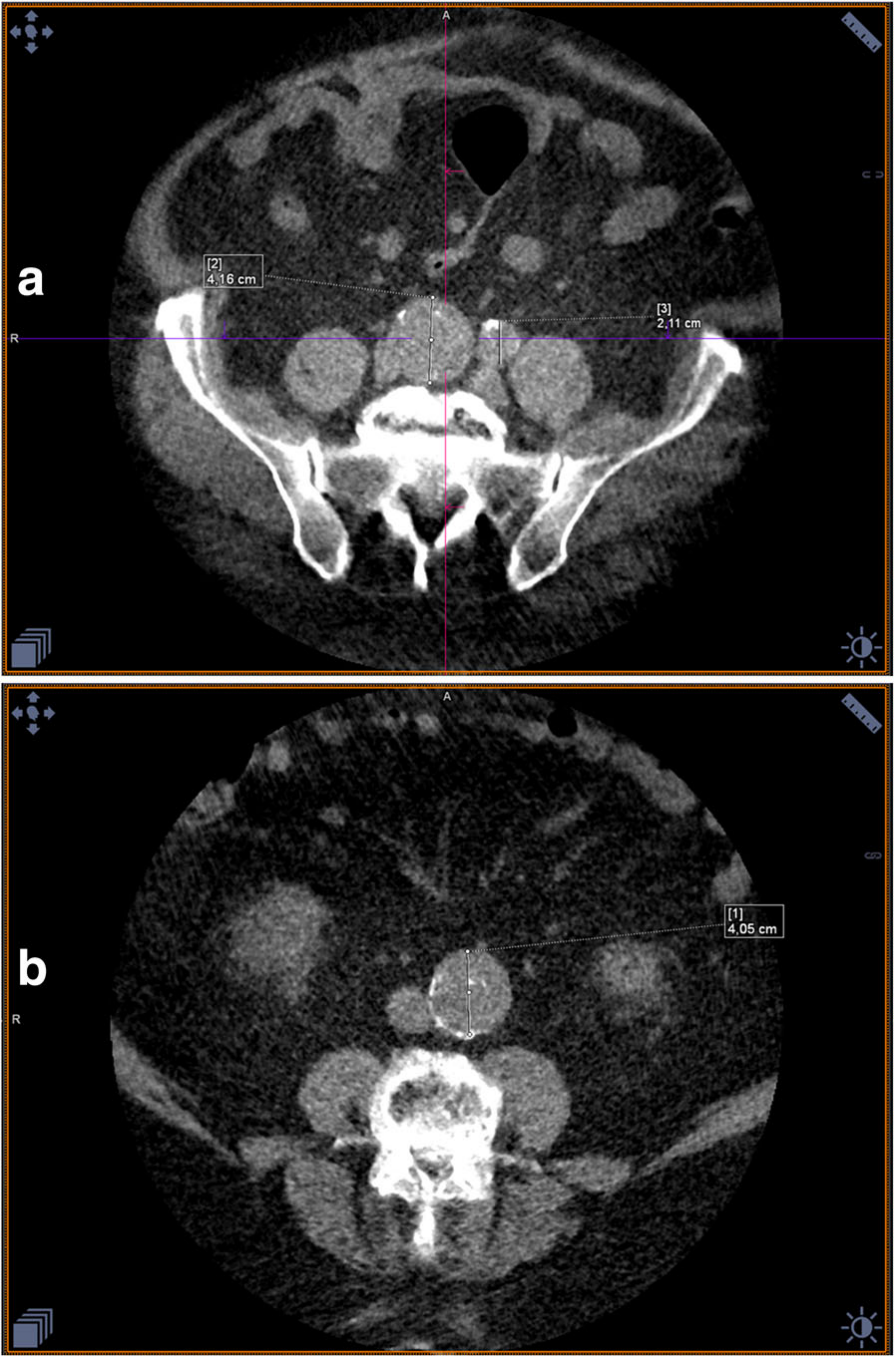
**a** Shows AAA measured 41.6 mm and a.iliaca aneurisms measuring 21.1 mm. **b** False Negative US finding, was measured to be 25.7 mm in the transverse with US, however nCT measured it was found to be 40.5 mm

533 men, mean age 69.4 years ±2.51 (1SD), underwent *both* nCT and US, additionally 4 (0.7%) of these were unable to be assessed satisfyingly by US, due to adiposity and/or intestinal gas, these were excluded from the calculations completely. Two of the 4 US invisible cases had an AAA diagnosed by nCT sized 32 mm and 42 mm, respectively. Consequently, 529 underwent both nCT and US. Thirty AAA were discovered using nCT, resulting in an occurrence of 5.7% (95% CI: 4.2;7.5%). US failed to identify 9 of these aneurysms, which were measured to be 27.4–42.8 mm in AP and 27.3–40.5 mm in the transverse plane with nCT (Fig. 1a and b).

**Fig. 2:**
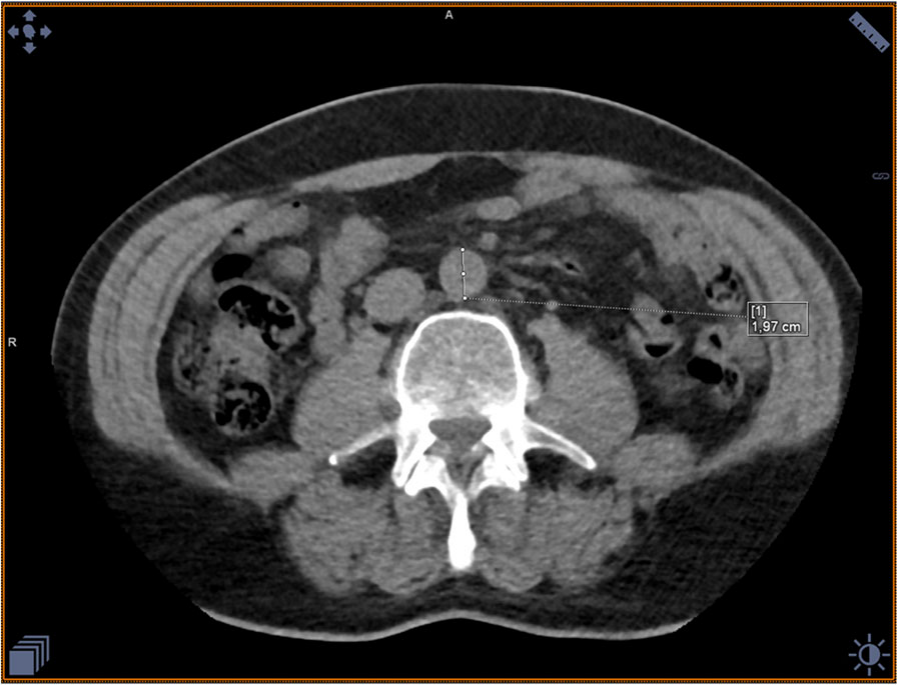
Transverse measurement done with CT was 19.7 mm, US grossly overestimated this at 44.6 mm. It should be mentioned that the participant in question was obese, making US examination troubling

US diagnosed 24 AAA (4.5% (95% CI: 3.0;6.6%)), 3 of which were not identified by nCT, these were found to be 30.2–31.8 mm in AP plane and 19.8–44.6 mm in the transverse plane using US, these were however measured by nCT to range from 18.3–19.7 mm in both planes (Fig. 2).

Unfortunately, the US examinations were not stored, but the CT scans were. Two senior consultants reexamined the nCT scans of the 12 conflicting findings blinded by knowledge of which test modality was used to diagnose the aneurysm. They uniformly classified all the 9 cases only diagnosed by nCT as AAA, and none of the 3 AAA diagnosed by US scans.

In addition, 5 isolated iliac aneurysms (≥20 mm) (0.9%, 95% C.I.: 0.3;2.2%) were discovered by nCT, − none of these were discovered by US, which were also validated by senior consultants.

### Sensitivity, and specificity and predictive values

Each modality was used as a reference standard for the other to analyze sensitivity and specificity respectively. Iliac aneurysms were not included as positive findings, when calculating sensitivity and specificity.

US performed with a modest sensitivity ranging from 57.1% (95% C.I.: 37.2;75.5%) to 70.4% (95% C.I.: 49.8;86.3%), with high specificity ranging from 99.2% (95% C.I.: 97.9;99.8%) to 99.6% (95% C.I.: 98.6;99.9%) (Table 2).

nCT performed better with a sensitivity ranging from 82.6% (95% C.I.: 61.2;95.1%) to 88.9% (95% C.I.: 65.3;98.6%). Concerning specificity, nCT fared comparably to US with a specificity of 97.7% (95% C.I.: 95.9;98.8%) to 98.4% (95% C.I.: 96.9;99.3%) (Table 3).

**Table 2:** Sensitivity, Specificity and predictive values when US compared to nCT as reference standard CT

	Sensitivity	95% CI	Specificity	95% CI	PV+	95% CI	PV-	95% CI
AP	0.7037	0.5;0.86	0.9920	0.98;0.99	0.8261	0.68;0.97	0.9841	0.68;0.97
Trans	0.5714	0.37;0.76	0.9960	0.99;0.99	0.8889	0.65;0.98	0.9765	0.96;0.99
AAA	0.7000	0.51;0.85	0.9940	0.98;0.99	0.8750	0.68;0.97	0.9821	0.97;0.99

For each measured plane, the sensitivity and specificity values and their corresponding 95% CI interval is presented. Additionally, positive and negative predictive values are included, with their 95% CI interval

AP: Cases are participants with a anterior posterior mesurement of >30 mm

TRANS: Cases are participants with a transverse measurement of >30 mm

AAA: Cases are particpants with measurement in any plane of >30 mm

**Table 3:** Sensitivity, Specificity and predictive values when CT compared to US as reference standard^a^

	SENSITIVITY	95% CI	SPECIFICITY	95% CI	PV+	95% CI	PV-	95% CI
AP	0.8261	0.61;0.95	0.9841	0.97;0.99	0.7037	0.5;0.86	0.9920	0.98;0.99
TRANS	0.8889	0.65;0.99	0.9765	0.96;0.99	0.5714	0.37;0.76	0.9960	0.99;0.99
AAA	0.8750	0.68;0.97	0.9821	0.97;0.99	0.7000	0.51;0.85	0.9940	0.98;0.99

^a^AP : Cases are participants with a anterior posterior mesurement of >30 mm

TRANS: Cases are participants with a transverse measurement of >30 mm

AAA: Cases are particpants with measurement in any plane of >30 mm

Expert review in those cases where US found an aneurysm, and nCT however did not, resulted in nCT sensitivity of 100% (95% C.I: 88.4;100%) and equally with a specificity of 100% (95% C.I: 99.3;100%).

### Analysis of discrepancies concerning diameter

Comparing all measurements including AAA, mean diameters in CT^ap^ and US^ap^ measurements show means of 21.3 and 21.2 mm respectively, with standard deviations of 5.3 and 5.0 (paired mean difference −0.05 ± −3.16 (SD), *p* = 0.70). The same applies for the measurements for the transverse plane showing CT^trans^ and US^trans^ means of 21.6 and 21.3 mm respectively, along with standard deviations of 5.5 and 5.1 (paired mean difference −0.28 ± −3.67 (SD), *p* = 0.08).

**Fig. 3:**
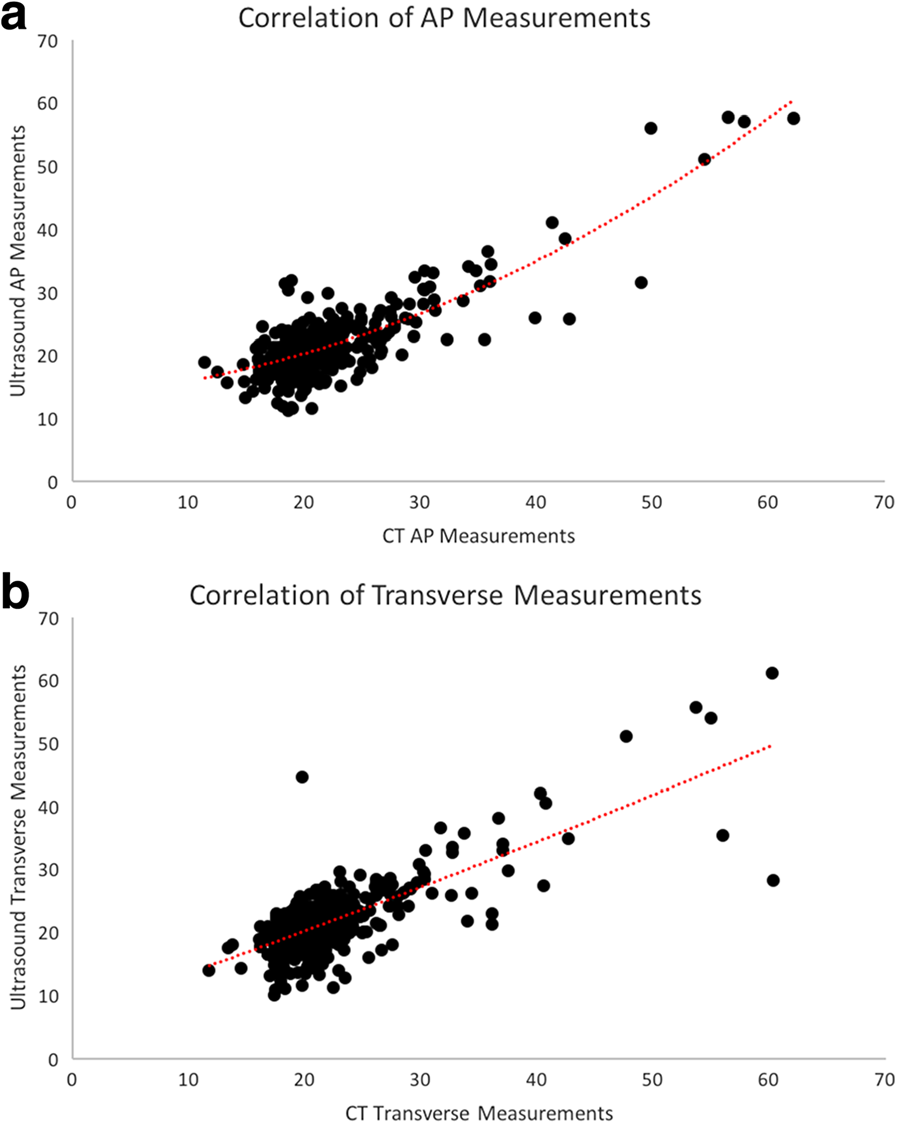
**a**, Correlation of *all* AP measurements. X-axis show the mean CT AP measurements. Y-axis show mean US AP measurements. **b**, Correlation of *all* Transverse measurements. X-axis show the mean CT transverse measurements. Y-axis show mean US transverse measurements

**Fig. 4:**
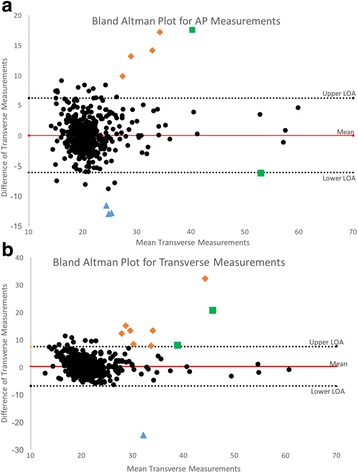
**a**, Title: Bland Altman Plot for AP measurements. x-axis represents the aortic measurements size, with the y-axis presenting the difference of measurements between the utilized modalities. Legend: Diamond : Classified as AAA by nCT not by US. Square: Both classified as AAA. Triangle: classified by US as AAA only. **b**, Title: Bland Altman Plot for transverse measurements. x-axis represents the aortic measurements size, with the y-axis presenting the difference of measurements between the utilized modalities. Legend: Diamond : Classified as AAA by nCT not by US. Square: Both classified as AAA. Triangle: classified by US as AAA only

Pearson’s correlation analysis of the measured diameter by the two modalities showed good agreement concerning AP measurement (Rho = 0.81, *p* < 0.0001) and to a close extent concerning transverse measurements (Rho = 0.75, *p* < 0.0001) (Fig. 3a and b). Bland-Altman plots [14] presenting the difference vs. the mean of the measured diameter in both planes showed apparently, no tendency to increasing difference with increasing diameter in either planes (Fig. 4a and b). However, Pearson’s correlation analysis of the difference versus the mean diameter was r = 0.114 (*p* = 0.0088) concerning AP measurements, and r = 0.083 (*p* = 0.0569) concerning transverse diameter indicating a minor increasing difference by increasing maximal aortic diameter in both planes.

**Fig. 5:**
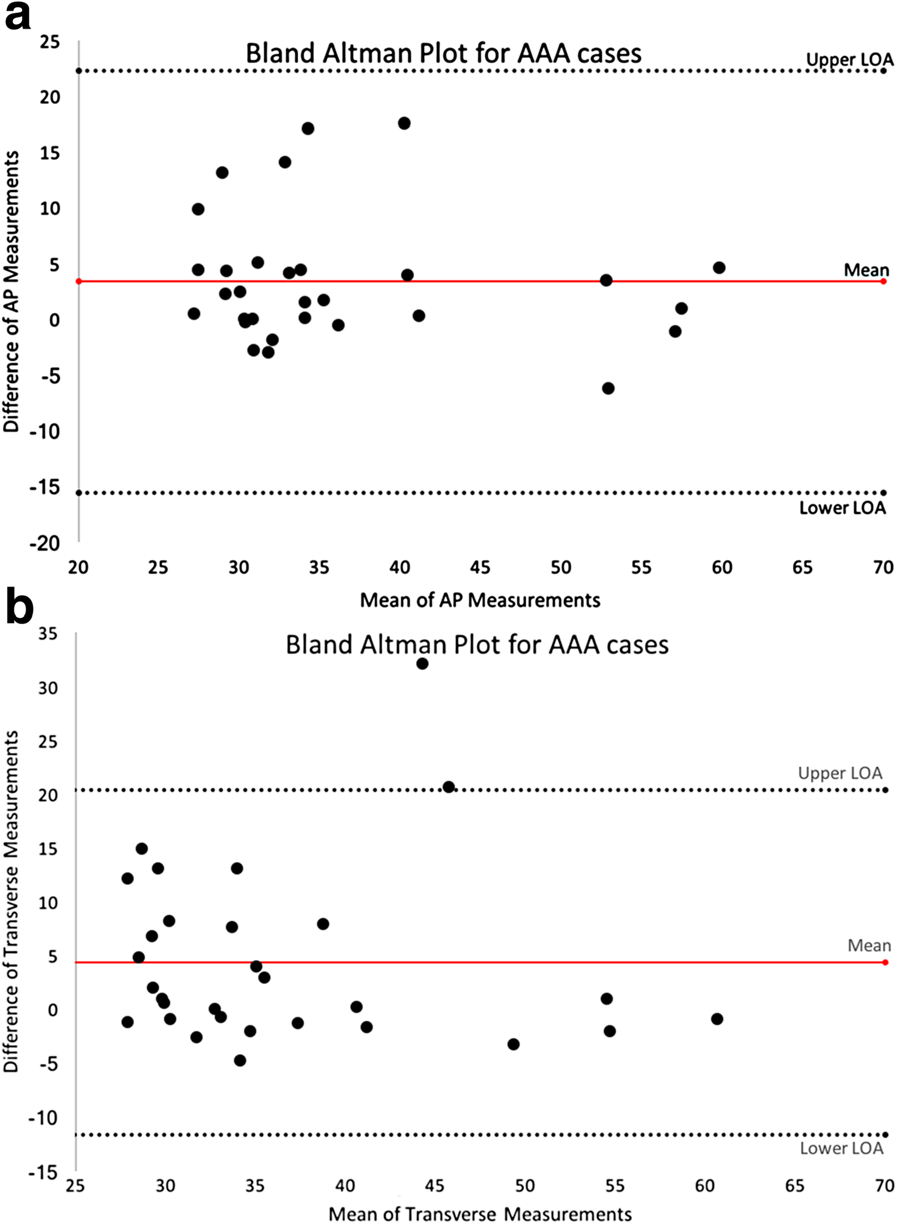
**a**, Title: Bland Altman Plot for AAA cases AP. x-axis show the mean aortic AP measurements, with the y-axis showing the difference of measurements between the utilized modalities, these represent AAA cases only. **b**, Title: Bland Altman Plot for AAA cases Transverse. x-axis show the mean aortic transverse measurements, with the y-axis showing the difference of measurements between the utilized modalities, these represent AAA cases only

Comparing mean AAA diameters in CT^ap^ and US^ap^ measurements show means of 38.1 and 34.7 mm respectively, with standard deviations of 9.7 and 10.5 (paired mean difference −3.3 ± 5.8 (SD), *p* = 0.004). The same applies for the measurements for the transverse plane showing CT^trans^ and US^trans^ means of 38.6 and 34.2 mm respectively, along with standard deviations of 9.5 and 9.9 (paired mean difference −4.39 ± 8.17 (SD), *p* = 0.006). Pearson correlation analysis of the measured diameter by the two modalities showed only a modest agreement concerning AP (r = 0.7508, p < 0.0001) and transverse measurements (Rho = 0.7008, p < 0.0001). Pearson correlation analysis of the difference versus the mean diameter was Rho = 0.1853 (p < 0.0001) and r = 0.1203 (*p* = 0.0055) concerning AP and transverse diameter, respectively. Bland-Altman plots examining the recorded AAA cases, showed increased difference between the used modalities with increasing diameters (Fig. 5a and b).

## Discussion

This is the first direct comparison of screening for AAA with non-contrast CT versus US. nCT was found to have superior sensitivity compared to US, and similar specificity. Our study is hampered by the lack of a real reference modality such as contrast CT, or contrast MRi. However, this was not included in the primary protocol because of the lack of feasibility to include such a modality. It was therefore decided that the modalities would be held up against each other, as reference standards, since neither had been validated as a AAA screening modality.

This study shows that in a screening setting, nCT has improved sensitivity over US. However, there is still a great deal of clinical evidence favoring US as a method, due to the reduced costs availability, and high specificity.

When aorta is visible utilizing US, it showed reasonable sensitivity for US with nCT being superior over US. Both modalities had a comparable high specificity. In addition, isolated iliac aneurysms are not likely to be detected by US, because AAA screening does not include the iliac arteries when using US. Consequently, as a screening tool for AAA, nCT seems acceptably valid, which is coherent with our hypothesis. In addition, it adds to the shortcomings of current AAA screening programs, because it is able to include the iliac arteries as well. Whether it too is acceptable as part of a multifaceted screening offer, we cannot conclude, as re-invitations, and final attendance rates are not yet available. It should be noted, that the pilot study was troubled by some preventable mishaps, with random lacking ultra sound devices, and not being able to review the US images being the most important issues. However, these issues would probably not have changed the final results of this study, but are worth mentioning. A possible limitation of this study is the lack of a truly accurate reference standard, which in this case would be 3D contrast enhanced CT scans, but this was not feasible nor ethically responsible to include.

Only men were invited to participate in this study, and this could be argued as a limitation, however, men are at increased risk solely because of their gender why a cardiovascular screening program would be targeted at men. However, a subgroup of women will be invited, to evaluate the potential cost-benefit of expanding the screening program to include women.

Although, nCT showed a comparable specificity to US, we cannot conclude that this should be the reference standard for screening for AAA as it is not widely available causing longer travel distances with assumable lower attendance, is time consuming and thus expensive. Nevertheless, nCT was able to detect more AAA (prevalence 5.7% versus 4.5%) and iliac aneurysms compared to US. This could – at least partially – explain the relatively low reduction in aneurysm related death in US-based randomized screening trials. nCT may thus be more efficient and perhaps a cost-effective alternative in a screening scenario, this however requires more data than currently available. This is especially true if repeated US scans are required to improve sensitivity to a comparable level of CT, since only one repetition of a US scan, closes the cost-gap between US and CT.

The medical students were trained in US, but have not undergone the same magnitude of screening as ultra-sonographers and other health care personnel conducting AAA screening. On the other hand, equipment with better-quality resolution, than portable scanners can offer was used. IAD was measured outer-to-outer, to be comparable to the UK screening program which also measures IAD in this fashion. The majority (7 of 9) of the AAA not detected by US but having visible aortas were found to be ectatic (>25 mm), while two were normal < 25 mm. These might have been detected by later 5-year interval, if introduced, since half of ectatic cases develop true AAA within 5 years [2]. This is due to true incidental cases or false negative findings. Those detected as ectatic by US but positive with nCT may be false positives, this could question whether rescreening five years after non-contrast screening will be beneficial [18]. However, they hardly make out the 50% reported to develop an AAA in this subgroup of the male population [2]. Consequently, DANCAVAS will re-invite this group after five years. Additionally, it could be argued that the 3 cases found by US and not by nCT, are actually false positives, thus making nCT appear less precise than it essentially is.

While US is an acceptable screening modality, it does have some shortcomings, mainly patients with a large waist circumference or intestinal gas diameters become difficult to asses properly, there are of course certain maneuvers to improve the assessment, but in a screening scenario these are not feasible.

We theorized that calcification would improve the validity of the non-contrast CT, but have not recorded any aortic calcification quantification. Consequently, we used two indirect signs of calcification as the coronary artery calcification score and ankle brachial index. The coronary artery calcification score correlated significantly positively with the difference of the measurements. However, this could be due to confounding from a clear positive correlation between coronary artery calcification score and waist circumference, as the other indirect calcification marker, lowest measured ankle brachial index, did not correlate with the observed differences.

As an epidemiological sub finding, this study also gave a modern estimate on the prevalence of AAA in Denmark in men, which does not seem to decline as reported in UK and Sweden [19]. The prevalence of AAA in Denmark remains relatively high. US based prevalence on Fyn (DANCAVAS 2014) is almost similar to the prevalence of 4.2% detected in the Viborg County (1994–98) [20] and higher than the prevalence of 3.3% detected in the VIVA trial (2008–11) in the Mid region of Denmark [21]. However, it should be noted that this is a small sample, and as the DANCAVAS trial continues, the AAA prevalence will be reported with increased certainty.

Using low dose nCT for screening purposes will ultimately result, in increased radiation exposure to those participating. However, screening for AAA is a one-time event, which in combination with the advances made with modern CT-scanners reduces this risk greatly, making the risk negligible in these elderly males [22]. Thus, making nCT a worthwhile modality, since it allows for a more thorough CVD screening than US does, while not inducing illnesses. Additionally, there may be incidental findings further improving disease prevention, this however would require the participant’s approval. This was not a part of this study, however, if a suspicious found was made by accident, the participant was informed and referred to the relevant specialties.

It is worth noting, that there is a secondary benefit to a reliable screening method, because of the psychological impact a false positive or negative result will have on the participant. This is especially important, when screening for common and potentially lethal diseases.

## Conclusions

Low-dose nCT scanning seems to be more sensitive than US, screening for AAA, making it a possible tool for a larger scale screening program.

Expanding the screening to not only include AAA but also generally for CVDs, nCT may become truly beneficial, because it enables evaluations of the aortic and iliac vessels in their entire length, as well as evaluating any arcane lesions to the coronary arteries, thus providing more information about the patient’s possible risks – this however requires additional research.

## Abbreviations

AAA: Abdominal Aortic Aneurysm
AP: Anterior-Posterior
CVD: Cardiovascular disease
DANCAVAS: The randomized Danish CardioVascular Screening trial
IAD: Infrarenal aortic diameter
nCT: Non-contrast-enhanced computed tomography
US: Ultrasound
